# Mutations in *PGRN* gene associated with the risk of psoriasis in Pakistan: a case control study

**DOI:** 10.1186/s12920-023-01757-8

**Published:** 2023-12-21

**Authors:** Saima Saleem, Zunaira Imran, Azam Samdani, Bahram Khoso, Sitwat Zehra, Abid Azhar

**Affiliations:** 1 The Karachi Institute of Biotechnology and Genetic Engineering (KIBGE), University of Karachi, Karachi, Pakistan; 2 Department of Dermatology, National Medical Centre (NMC), Karachi, Pakistan; 3 Department of Dermatology, Jinnah Sindh Medical University (JSMU), Karachi, Pakistan

**Keywords:** Psoriasis, PGRN gene, Single nucleotide polymorphisms, Pathogenesis, Inflammatory cytokines

## Abstract

**Background:**

Psoriasis is a chronic, autoimmune, papulosquamous skin disorder, characterized by the formation of drop-like papules and silvery-white plaques surrounded by reddened or inflamed skin, existing predominantly on the scalp, knees and elbows. The characteristic inflammation and hyperproliferation of keratinocytes in psoriasis is regulated by progranulin (PGRN), which suppresses the expression and release of inflammatory cytokines, such as TNF-α.

**Methodology:**

In this study mutation analysis of the *PGRN* gene was performed by extracting the genomic DNA from blood samples of 171 diagnosed psoriasis patients and controls through standard salting-out method, followed by amplification and sequencing of the targeted region of exon 5–7 of *PGRN* gene.

**Results:**

Three single nucleotide polymorphisms, rs25646, rs850713 and a novel point mutation 805A/G were identified in the *PGRN* gene with significant association with the disease. The variant alleles of the polymorphisms were significantly distributed among cases and controls, and statistical analysis suggested that the mutant genotypes conferred a higher risk of psoriasis development and progression. Multi-SNP haplotype analysis indicated that the CAA (OR = 8.085, 95% CI = 5.16–12.66) and the CAG (OR = 3.204, 95% CI = 1.97–5.21) haplotypes were significantly associated with psoriasis pathogenesis.

**Conclusions:**

These findings demonstrate that polymorphisms in *PGRN* might act as potential molecular targets for early diagnosis of psoriasis in susceptible individuals.

## Background

Psoriasis, a chronic autoimmune inflammatory skin disorder, is one of the leading skin conditions with a global prevalence ranging from 0.09 to 11.4% [1]. The disease is generally characterized by erythematous, sharply demarcated, symmetrical papules or plaques covered with thick silvery-white scales present mainly on the knees, elbows, scalp, nails, umbilicus and the lumbar region [2–5]. Psoriasis is known to negatively impact the patient’s quality of life (QoL) and its pathogenesis is mainly influenced by genetic predisposition and environmental factors, such as physical trauma, infections, smoking and alcohol consumption [6–8]. While psoriatic arthritis, psychological stress, obesity and metabolic syndrome, as well as cutaneous malignancies are the comorbidities associated with severe psoriasis [4, 9].

The disease presents five distinct clinical manifestations, i.e. Psoriasis Vulgaris (commonly referred to as plaque psoriasis) which is characterized by the appearance of reddened, inflamed skin with silvery-white plaques, Guttate psoriasis exists in the form of drop-like papules, while small pustules appear on the palms and feet of Pustular psoriasis patients. Erythrodermic and Inverse are the rare forms of psoriasis characterized by skin shedding and sharply demarcated, wet plaques respectively. The severity of the disease varies among patients, and greatly depends on the extent of exposure to triggering factors.

**Fig. 1:**
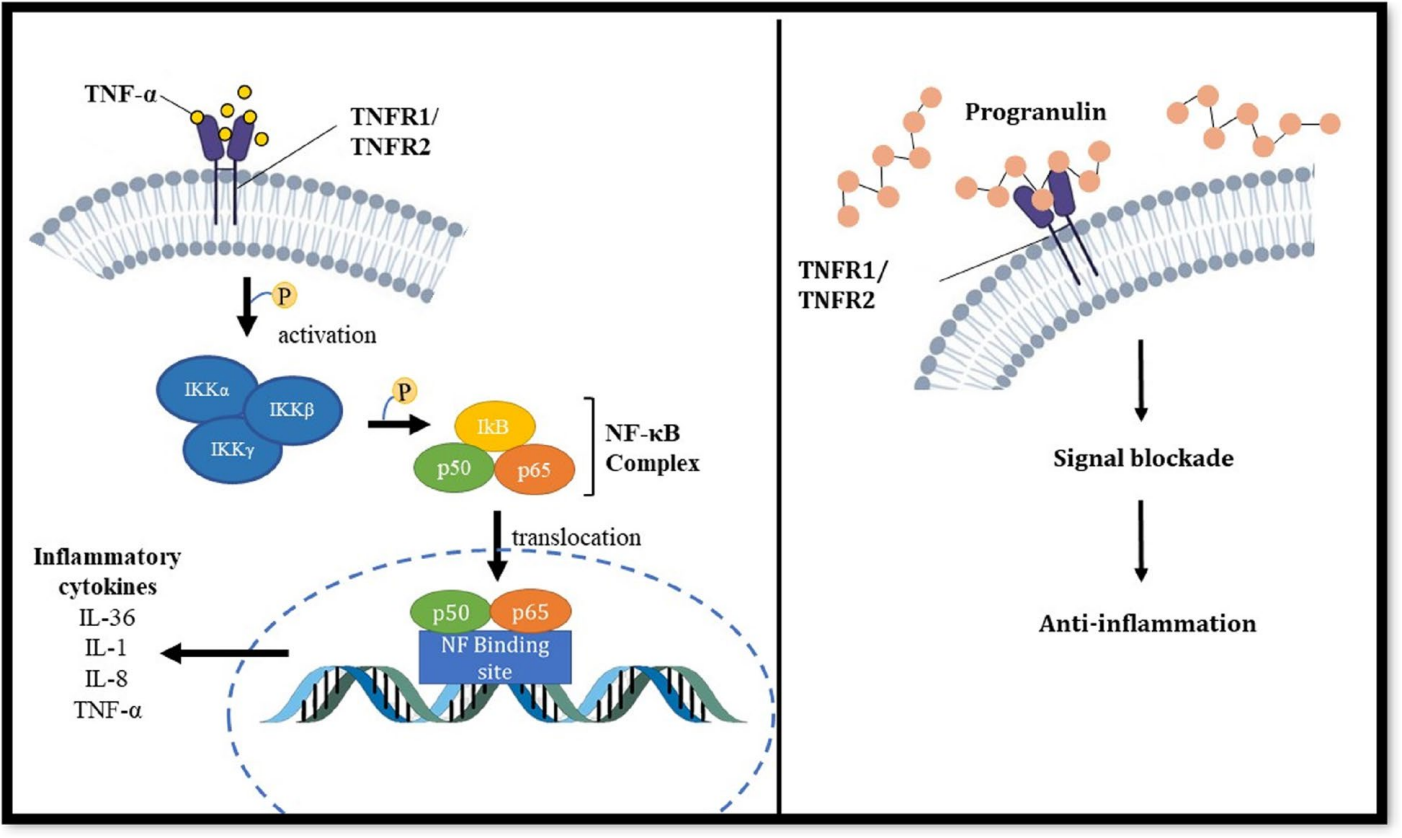
The molecular pathway of TNF and Progranulin in immune regulation. The binding of TNFα with TNFR1/2 stimulates the phosphorylation of inhibitor of kappa B kinase (IKK), leading to the activation and translocation of NF-κB into the nucleus resulting in synthesis and subsequent release of inflammatory cytokines (IL-36, IL-1, IL-8). Three domains of progranulin bind to the TNFR1 and regulate inflammation by disrupting the TNFα/NF-κB signalling pathway [17]

This dis-figuring disease is known to be activated by the innate immune system that releases pro-inflammatory cytokines, eventually leading to distinct epidermal and vascular hyperplasia which is a key characteristic of lesional psoriatic skin [10–12]. One of the key inflammatory cytokines observed to be in elevated levels in psoriasis is tumor necrosis factor α (TNF-α) which binds to its specific receptors, TNFR1 and TNFR2, and stimulates the nuclear factor kappa B (NF-κB) signaling pathway to initiate rapid transcription of other inflammatory genes such as IL-6, IL-8, IL-1β and INF-γ, as depicted in Fig. 1 [3, 13–16].

A TNF antagonist, Progranulin, which also serves as a growth factor protein, inhibits the TNF-α pathway by competitively binding to TNFRs (Fig. 1) [18–22]. Overexpression of Progranulin (encoded by the *PGRN* gene, located at chromosome 17 - NC_000017.11 (44,345,302..44353106)) in the epidermal keratinocytes of psoriasis patients, and elevated serum levels in patients with lesional psoriatic skin have suggested a therapeutic role of progranulin in regulating inflammation [13, 23, 24]. Progranulin is implicated in various cellular processes which include cell growth, wound healing, cartilage development, mediating inflammation, is a well-known biomarker for different cancers, and its deficiency has been linked with various neurological diseases [24, 25].

Inhibiting the TNF-α signaling pathway by blocking its receptors has the potential to induce an anti-inflammatory response in inflammatory psoriatic skin [25–28]. Hence, this study aims to contribute to the existing knowledge of disease pathogenesis by identifying genetic markers involved in the onset and aggravation of psoriasis, and investigate the loss of function single nucleotide polymorphisms in the *PGRN* gene that may alter the activity of progranulin protein and modify its anti-inflammatory potential.

## Methods

### Subject selection and sample collection

The present study comprised of 342 subjects, including 171 psoriasis patients and 171 healthy individuals as controls. Patients diagnosed with any of the five subtypes of psoriasis were included in the study while patients diagnosed with bacterial or fungal skin infections were excluded, whereas the control group consisted of healthy individuals with no history of skin disorders. A questionnaire was employed to collect the clinical details of each patient and all subjects were recruited with a written informed consent. Ethical approval for this case-control study was taken from the Institutional Review Board (IRB) of The Karachi Institute of Biotechnology and Genetic Engineering (KIBGE), and a collaboration was established with Jinnah Post Graduate Medical Centre (JPMC). Blood samples were collected from the study subjects and stored at 4 °C in vacutainers containing the anticoagulant ACD (Acid Citrate Dextrose).

### DNA extraction and quantification

Genomic DNA was extracted from peripheral blood leukocytes by following the standard salting-out protocol [29]. The quality of the extracted DNA was assessed through agarose gel electrophoresis using 0.8% agarose, while the concentration and purity of the DNA were determined through nano-drop spectrophotometer (Thermo Fisher Scientific, Waltham, MA, USA).

### Primer designing and PCR amplification

The sequence of the *PGRN* gene (ENSG00000030582) was retrieved from the *Ensemble Genome database.* The exome 5–7 was selected for analysis due to a higher frequency of variants that have been linked with other diseases, as cited in the dbSNP. The relative forward and reverse primers for the polymerase chain reaction were constructed using an online *Primer 3* software. The specificity of the designed primers was checked by *Primer-BLAST* software.

PCR amplification of the targeted region (from exon 5 to 7) of the *PGRN* gene was carried out using 2 μL of 50 ng/μL DNA and 0.1 μL each of 20 μM forward (5′-CACCAGCTCCTTGTGTGATG-3′) and reverse (5′-TGGTAGCGTTCTCCTTGGAG-3′) primers. The total volume of each reaction was 20 μL. Amplification was performed using the Molequle-On Thermal Cycler (MOLEQULE-ON, Auckland, New Zealand), in which each reaction was subjected to 94 °C for denaturation and annealing at 61.8 °C, with extension at 72 °C. The obtained products were run on 2% agarose gel alongside a 1 kb marker at 100 V for 40–45 min. The DNA bands were observed on the FastGene® FAS V gel documentation system (Nippon genetics, Germany).

### Sequence analysis

The PCR products obtained were purified from the gel via MQ Gel Purification Kit (MOLEQULE-ON, Auckland, New Zealand). The purified products were sequenced via Sanger sequencing technique and were analysed by MEGA X software.

### Statistical analysis

The association of SNPs between cases and controls were analysed by Pearson’s Chi-square test. Genotypic and allelic frequencies were determined by direct counting. MedCalc (https://www.medcalc.org/calc/odds_ratio.php), an online statistical tool, was employed to calculate the odds ratio (OR) at a 95% confidence interval (CI). The linkage disequilibrium plot and haplotype frequencies were estimated by the SHEsis program (http://shesisplus.bio-x.cn/SHEsis.html). Genetic models such as dominant, co-dominant, over dominant and recessive models were also tested to determine the genotypic and allelic associations of the identified polymorphisms with susceptibility to psoriasis.

## Results

A total of 342 diagnosed psoriasis patients and controls were genotyped in this study. Among the patients, 72% were plaque psoriasis patients, 12% suffered from pustular, 11% from erythrodermic and 5% patients were from nail psoriasis. According to our data, the highest number of psoriasis cases emerged in the age group of 21–30 years. A detailed account of the baseline demographic characteristics of patients and controls is given in Table 1.

**Table 1:** The baseline characteristics of Psoriasis patients and controls

	Patients	Controls
	*n* = 171	*n* = 171
Sex		
Males	(102) 59.65%	(106) 61.99%
Females	(69) 40.35%	(65) 38.01%
Age, median ± SD (years)	29 ± 14.98	30 ± 12.94
Family history, %	(48) 28%	
Psoriasis classification		
Plaque	(123) 72%	
Erythrodermic	(19) 11%	
Pustular	(21) 12%	
Nail	(8) 5%	

**Fig. 2:**
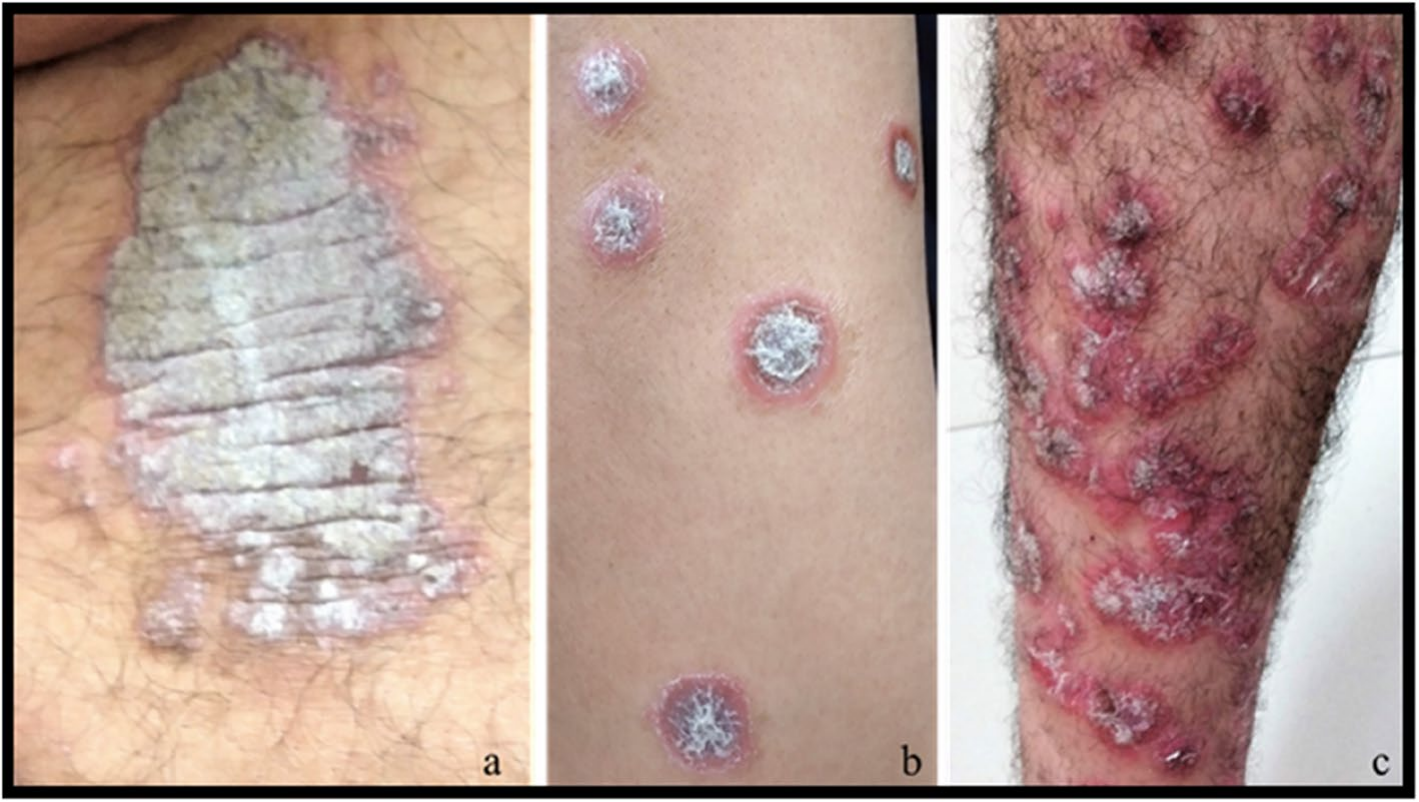
Psoriasis vulgaris, commonly termed as plaque-type psoriasis, exists in varying clinical forms. **a** Chronic plaque psoriasis with thick white plaque on the lower back. **b** Symmetrical lesions distributed non-uniformly on the fore-arm. **c** Erythematous and extensively confluent plaques on the leg

Although plaque psoriasis was the most prevalent type, some variations were observed in the clinical presentation of the disease among patients. Many patients exhibited large, asymmetrical lesions with thick white plaques, on their knees, elbows and lower backs (Fig. 2a), while some patients demonstrated small, circular lesions with thin silver plaques distributed non-uniformly on their arms and legs (Fig. 2b and c).

### PGRN gene polymorphisms

**Fig. 3:**
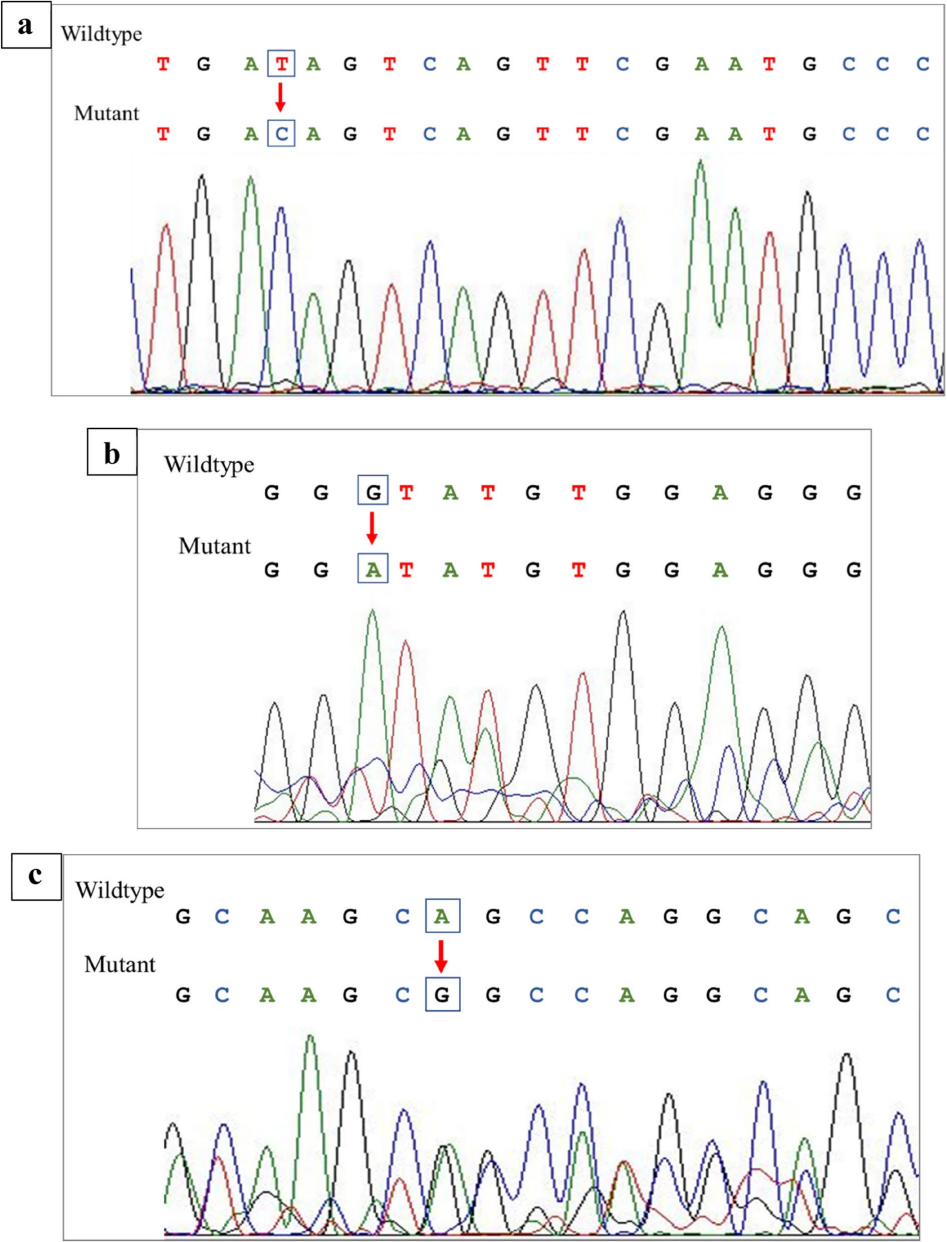
Electropherograms indicating change in nucleotide sequence of PGRN gene. **a** A single blue peak at exon 5 indicates change of nucleotide from T to C (rs25646). **b** A change of nucleotide from G to A observed in rs850713. **c** A novel point mutation observed at intron 7 (805 A/G). The double peak observed indicates heterozygosity

Amplification of the exon 5–7 of the *PGRN* gene produced a single band on the agarose gel, with a product size of 978 bp, which was purified for sequence analysis. Two *PGRN* variants, rs25646 and rs850713, were identified in psoriasis patients, as indicated in Fig. 3. In addition, an unknown point mutation, c.805 A/G, located at intron 7 was also identified (Table 2).

**Table 2:** Summary of variants of PGRN gene in psoriasis patients and controls

SNPs	Chromosome position	SNP property	MAF
rs25646	17: 44350262	Exon 5	0.08546
rs850713	17: 44350364	Intron 5	0.2537
805 A/G	17: 44350897	Intron 7	–

Genomic DNA from psoriasis patients was used to amplify the 978 bp DNA fragment covering the targeted region of *GRN* gene. DNA sequencing was performed, and two *PGRN* SNP sites and one point mutation were identified

### Frequency distribution

Genotype distribution of the *PGRN* polymorphisms between psoriasis patients and controls is presented in Table 3. The homozygous mutant CC genotype of rs25646 was highly prevalent in patients as compared to the controls, and the wildtype T allele was present abundantly in controls while the mutant C allele was abundant in patients.

**Table 3:** Genotypic frequencies and association analysis of SNPs among psoriasis patients

SNP	χ^2^	Odds Ratio (95% CI)	*p* value	Genotype	Patients [n (%)]	Controls [n (%)]
rs25646	82.61	6.217 (3.34–11.57)	*p* < 0.001	TT TC CC	45 (26.32) 30 (17.54) 96 (56.14)	109 (63.74) 44 (25.73) 18 (10.53)
rs850713	88.21	6.668 (3.51–12.66)	*p* < 0.001	GG GA AA	50 (29.24) 33 (19.30) 88 (51.46)	136 (79.53) 6 (3.51) 29 (16.96)
805 A/G	25.18	2.875 (1.20–6.88)	*p* < 0.001	AA AG GG	105 (61.40) 63 (36.84) 3 (1.75)	146 (85.38) 24 (14.04) 1 (0.58)

For rs850713, the homozygous variant AA genotype was higher among patients, while the wildtype GG genotype was observed in a majority of controls. The allelic distribution showed that the mutant A allele had a higher frequency in cases, whereas the G allele was higher in controls. The novel variant, 805A/G existed mostly in heterozygous condition (AG genotype) in 36.8% of cases and 14% of controls.

### Association analysis

Pearson’s chi square test revealed that rs25646 and rs850713 were associated with the disease (Table 3). The odds ratio values indicated that rs25646 (*p* <  0.001, OR = 6.217, 95% CI = 3.34–11.57), rs850713 (*p* <  0.001, OR = 6.668, 95% CI = 3.512–12.66) and the unreported SNP 805A/G (*p* = 0.017, OR = 2.875, 95% CI = 1.201–6.883) were significantly associated with the risk of disease.

### Genetic model and linkage analysis

Genetic models identify the potential role of genotypes with respect to disease pathogenesis. Genetic modelling was performed for each genotype observed, and OR was calculated to evaluate the impact of each allelic combination on disease onset. The genotype association analysis of rs25646 showed that the CC genotype in dominant, recessive and codominant forms was linked with an increased risk of disease (Table 4). Similarly, the AA genotype of rs850713 was significantly associated with the risk of disease in dominant and codominant conditions. For the novel SNV, the heterozygous AG genotype was associated with an increased risk of psoriasis in codominant and over-dominant conditions.

**Table 4:** The analysis of inheritance mode for the genotypes of SNPs between psoriasis patients and controls

Genetic models	Genotype	OR [95% CI]	*p* value
**rs25646**			
Dominant	TT	1 (Ref)	< 0.001
	TC + CC	4.92 (3.10–7.81)	
Recessive	TT + TC	1 (Ref)	< 0.001
	CC	10.88 (6.13–19.32)	
Co-dominant	TT	1 (Ref)	0.08
	TC	1.65 (0.93–2.95)	< 0.001
	CC	12.92 (7.01–23.81)	
Over dominant	TT + CC	1 (Ref)	0.07
	TC	0.61 (0.36–1.04)	
**rs850713**			
Dominant	GG	1 (Ref)	< 0.001
	GA + AA	9.40 (5.72–15.45)	
Recessive	GG + GA	1 (Ref)	< 0.001
	AA	5.19 (3.15–8.55)	
Co-dominant	GG	1 (Ref)	< 0.001
	GA	14.96 (5.91–37.85)	< 0.001
	AA	8.25 (4.86–14.03)	
Over dominant	GG + AA	1 (Ref)	< 0.001
	GA	6.57 (2.68–16.15)	
**805 A/G**			
Dominant	AA	1 (Ref)	< 0.001
	AG + GG	3.67 (2.17–6.20)	
Recessive	AA + AG	1 (Ref)	0.34
	GG	3.036 (0.31–29.47)	
Co-dominant	AA	1 (Ref)	< 0.001
	AG	3.65 (2.14–6.219)	0.21
	GG	4.171 (0.43–40.66)	
Over dominant	AA + GG	1 (Ref)	< 0.001
	AG	3.573 (2.09–6.08)	

**Fig. 4:**
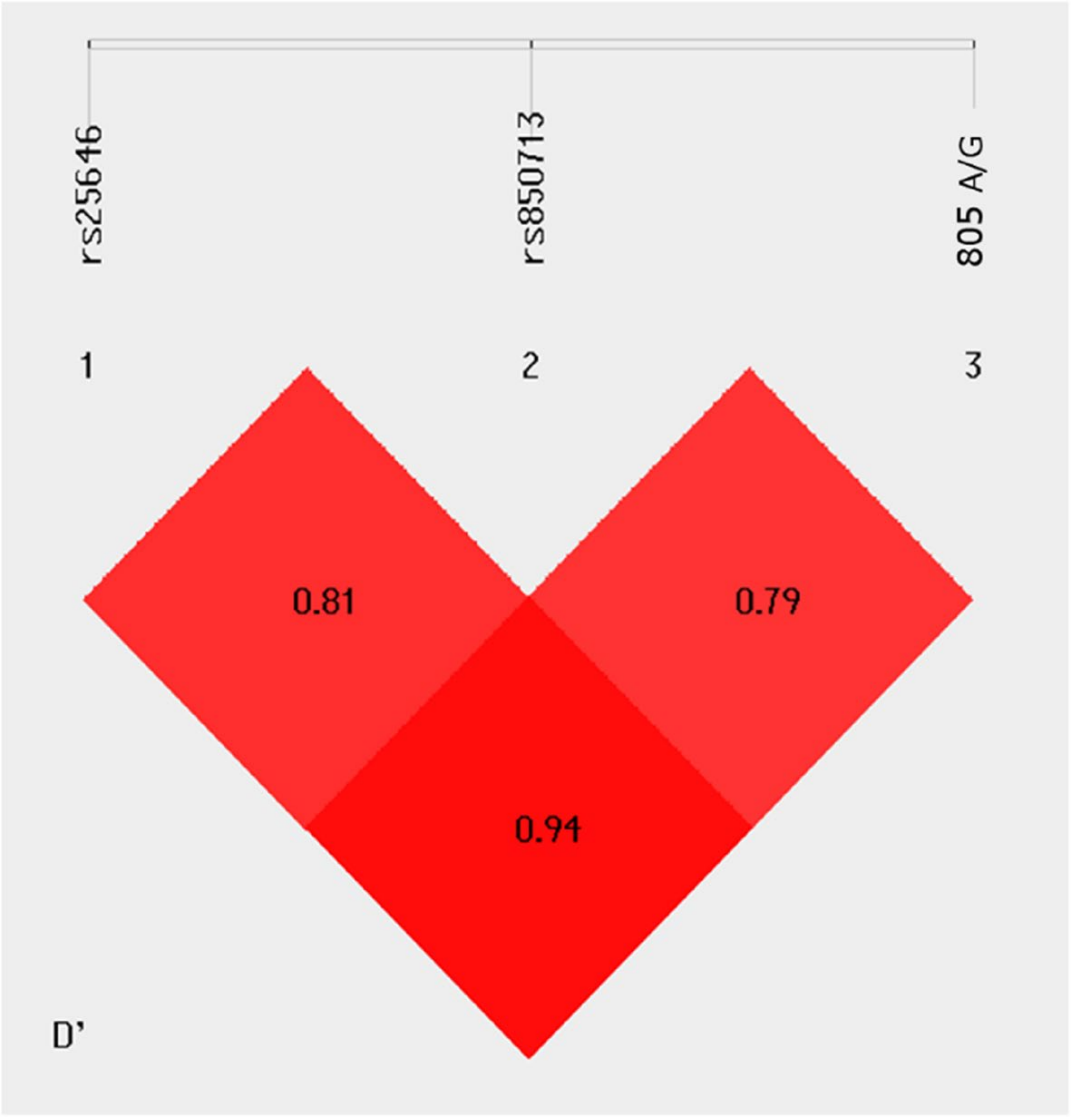
Linkage disequilibrium analysis for rs25646, rs850713 and the point mutation 805 A/G

The multi-SNP association analysis between rs25646, rs850713 and the point mutation is indicated in Table 5. Odds ratio confirm the role of CAA (OR = 8.085, 95% CI = 5.16–12.66) and the CAG (OR = 3.204, 95% CI = 1.97–5.21) haplotypes with the risk of psoriasis progression. While the TGA (OR = 0.19, 95% CI = 0.14–0.26) and the CGA (OR = 0.46, 95% CI = 0.23–0.91) haplotypes showed a protective effect among patients. The linkage disequilibrium plot suggested that the SNPs were in strong LD, and are likely to be inherited together (Fig. 4).

**Table 5:** Haplotypes of SNPs in patients and controls

Haplotype	Patients (freq)	Controls (freq)	χ^2^	*p* value	OR [95% CI]
TGA	120 (0.35)	253 (0.74)	104.3	*p* < 0.001	0.19 [0.14–0.26]
CGA	13 (0.04)	27 (0.08)	5.204	*p* < 0.05	0.46 [0.23–0.91]
CAA	140 (0.41)	27 (0.08)	101.15	*p* < 0.001	8.085 [5.16–12.66]
CAG	69 (0.20)	25 (0.07)	23.877	*p* < 0.001	3.204 [1.97–5.21]

Allelic combinations with a frequency lower than 3% in the subjects were excluded from analysis

## Discussion

Lesional psoriatic skin displays elevated PGRN levels in the epidermis and the infiltrating inflammatory cells, and overexpression of the protein suppresses the production of inflammatory cytokines, which include IL-1β, IL-6, COX-2 and IFN-γ [13, 23, 30]. Genetic variations in the *PGRN* gene can potentially dysregulate the anti-inflammatory potential, modify the expression pattern of the protein and may contribute to the initiation of diseases. In the current study, the polymorphisms rs25646 and rs850713 in the *PGRN* gene were investigated in diagnosed psoriasis patients and controls.

The SNP rs25646 (c.384 T/C), present on exon 5 of *PGRN,* is a missense mutation where the codon GAT is replaced by GAC. However, no change in the amino acid sequence is observed as both the codons code for aspartic acid (p.D128D). Genotypic analysis revealed that the mutant allele (C) existed frequently in cases (65%) and the wildtype T allele was frequent among controls (76%). However, a study conducted on the AS patients of the Chinese Han population reported contrasting results, where the mutant C allele was abundant in controls (16.7%) as compared to the cases (14.6%), thus suggesting that the SNP had no impact on AS susceptibility [25]. In the current study, the wildtype TT genotype was observed in 63.7% of the controls and 26% of cases, while the mutant CC genotype occurred in 56% of cases and 10.5% of controls. The heterozygous TC genotype was observed to exist more frequently in controls (25.7%) as compared to cases (17.5%) (Table 3). The SNP rs25646 was found to be significantly associated with the risk of disease development and progression. To further investigate the role of each genotype in the pathogenesis of psoriasis, genetic models were applied. It was observed that the dominant, co-dominant and recessive CC genotypes confer a higher risk of psoriasis among cases (Table 4). Whereas the TC genotypes were negatively associated with disease progression. Although no amino acid change was observed as the consequence of the studied SNP rs25646, such variations can disrupt the gene expression and may alter the structure and function of the protein [31], thus leading to disease development.

In this study, rs850713 also showed significant association with the risk of psoriasis onset and progression. This SNP results in the substitution of G to A. As denoted in Table 3, the frequency of the homozygous mutant genotype (AA) and the heterozygous GA genotype was higher in patients, 51.5 and 19%, as compared to the control subjects, 16.9 and 3.5% respectively. Comparable results were obtained in another study involving GD patients [19], where the GA genotype existed in 52% of the cases and 43% controls. The genetic models constructed for rs850713 (Table 4), suggested that GA and AA genotypes significantly contribute to the increased risk of developing psoriasis. These results predicted that the variant allele A is highly pathogenic and may have the potential to alter the splicing region and cause protein degradation, possibly due to misfolding.

Genetic analysis of the novel variant 805A/G revealed that the wildtype AA genotype was higher in frequency in both cases and controls, while the heterozygous AG genotype existed in 36.8% of cases and 14% controls and the homozygous mutant genotype (GG) was observed in 1.75% of patients and 0.6% controls. The distribution of 805A/G genotypes between diagnosed psoriasis patients and controls exhibited a significant association with the risk of disease development and progression in over-dominant and codominant AG genotypic forms. Whereas the GG genotype in recessive (AA + AG vs. GG) and codominant (AA + GG vs. AG) showed a significant negative association with psoriasis (Table 4). Thus, demonstrating that the G allele increases the risk of disease when it occurs in heterozygous form only. Although these variants seem benign, they may be involved in disease pathogenesis by altering the enhancer or silencer regions of the gene, thereby causing exon skipping leading to functional changes [32].

Previous reports highlighting the link between PGRN deficiency and psoriasis severity indicate that serum progranulin levels are negatively correlated with PASI scores [13]. Several studies have shown that serum and plasma PGRN levels are significantly lower in mutation carriers as compared to non-carriers [33]. SNPs can alter the expression of the *PGRN* gene and have been proposed as risk factors for neurodegenerative diseases such as frontotemporal lobar degeneration (FTLD) [34], Alzheimer’s [35], Gaucher’s [19] as well as tumor progression [24], inflammatory arthritis and psoriasis. However, Hu et al., [25] did not find a significant association between PGRN mutations and ankylosing spondylitis in the Chinese Han population.

Multi-SNP analysis between the three identified variants of *PGRN* gene exhibited that the CAA and CAG haplotypes were significantly associated with increasing the risk of psoriasis development and progression (Table 5). Linkage disequilibrium plot (Fig. 4) also verified that the detected SNPs, rs25646 and rs850713 were 81% (D′ = 0.81) associated, while the probability of inheriting the novel point mutation 805A/G with rs25646 and rs850713 were 94% (D′ = 0.94) and 79% (D′ = 0.79) respectively. These high associations are due to the presence of the variations on the same chromosome.

In light of the obtained results, the identification of *PGRN* gene polymorphisms provide a better understanding of the influence of gene variants on psoriasis pathogenesis. As the haplotype analysis demonstrated, the risk haplotypes may be useful in predicting the disease and its associated comorbidities in individuals at risk or have a history of psoriasis in their families. Similar studies exploring other regions of the gene coupled with expression analysis may help predict the role of *PGRN* as a possible biomarker for psoriasis.

## Conclusions

Progranulin plays a critical role as an anti-inflammatory agent by modulating the signalling pathways. The current study demonstrated the possible association of genetic variations (rs25646, rs850713 and the novel variant 805 A/G) in the *PGRN* gene with psoriasis pathogenesis. The CAA and CAG haplotypes were found to be significantly associated with the development of psoriasis. The identified *PGRN* gene variations may be involved in downregulation of the protein at serum level, thus interfering with the therapeutic potential of progranulin and increase the risk of developing susceptibility to psoriasis. Furthermore, the study suggests that genetic analysis of the *PGRN* gene in psoriasis patients may present a potential clinical approach for better screening, intervention and management of the disease.

## Data Availability

All data generated or analyzed during this study are included in this published article.
